# Neglect and Extinction Depend Greatly on Task Demands: A Review

**DOI:** 10.3389/fnhum.2012.00195

**Published:** 2012-07-17

**Authors:** Mario Bonato

**Affiliations:** ^1^Dipartimento di Psicologia Generale, Università di PadovaPadova, Italy

**Keywords:** awareness, cognitive resources, attention, dual-task, extinction, neglect, neuropsychology, computer-based testing

## Abstract

This review illustrates how, after unilateral brain damage, the presence and severity of spatial awareness deficits for the contralesional hemispace depend greatly on the quantity of attentional resources available for performance. After a brief description of neglect and extinction, different frameworks accounting for spatial and non-spatial attentional processes will be outlined. The central part of the review describes how the performance of brain-damaged patients is negatively affected by increased task demands, which can result in the emergence of severe awareness deficits for contralesional space even in patients who perform normally on paper-and-pencil tests. Throughout the review neglect is described as a spatial syndrome that can be exacerbated in the presence and severity by both spatial and non-spatial tasks. The take-home message is that the presence and degree of contralesional neglect and extinction can be dramatically overlooked based on standard clinical (paper-and-pencil) testing, where patients can easily compensate for their deficits. Only tasks where compensation is made impossible represent an appropriate approach to detect these disabling contralesional deficits of awareness when they become subtle in post-acute stroke phases.

## Neglect: Definition, Spatial (and Non-Spatial) Characteristics

Neglect is a disabling condition which often follows a brain lesion. Symptoms of neglect consist of the failure to report, respond, or orient to stimuli presented to the side opposite that of the damaged hemisphere (i.e., the contralesional hemispace), which cannot be explained by sensory-motor deficits (Heilman, 1979). According to this definition, any attentional deficit in contralesional processing which has an impact on everyday life, or on the experimental task performed by a patient with known – or suspected – brain damage can be, in the absence of an alternative explanation, attributed to neglect.

The characteristics of neglect change substantially in time. Its symptoms, striking and common in the acute phase, become less evident and frequent with time. In the first hours/days after the occurrence of a neurological insult (commonly, although not necessarily, a stroke) the presence of neglect is often self-evident in the form of the head and eyes deviating toward the ipsilesional space (Becker and Karnath, 2010; Karnath and Rorden, 2012). As time progresses, this deviation tends to decrease on its own. The presence and degree of neglect are then typically quantified according to the patients’ performance on specific paper-and-pencil tests, including cancellation (crossing of all target items within a sheet) and bisection (marking of the midpoint of a line) tasks.

Remarkably, the characteristics of neglect also change considerably according to the affected hemisphere. In the acute phase, neglect following right hemisphere damage is relatively more common than neglect following left hemisphere damage. In contrast, in the post-acute and chronic phases (left) neglect after right hemisphere damage is much more common and severe than (right) neglect after left hemisphere damage (Ringman et al., 2004; Stone et al., 1991, 1992, 1993). When considering patients with right hemisphere brain damage, the prevalence of neglect ranges from 13 to 82% (Bowen et al., 1999; Azouvi et al., 2002). This surprisingly high variability (see Barrett et al., 2006 for a thorough discussion) might depend on time from lesion onset, inclusion criteria and, crucially for the purposes of this review, on the heterogeneous methods used to diagnose neglect (e.g., number and complexity of tests, domain of space under investigation, see Azouvi et al., 2002). Critically, these factors can also interact in a dramatic manner. Recovery rates from acute neglect range from 60 to 90% within 3–12 months from the injury (Karnath et al., 2011). From these observations it might be concluded that the majority of patients with right hemisphere damage show neglect in the acute phase, and that many show a remission of the deficits in the chronic phase. However, it is possible that the perception of a “recovery” process may be illusory when based only on improved performance on paper-and-pencil tests, where patients can compensate for their deficits and hide the real extent of their impairment. In contrast, when testing procedures are adopted that do not allow patients to compensate for their deficits, apparently recovered patients often return to show severe contralesional deficits (Cherney and Halper, 2001; Robertson and Manly, 2002; List et al., 2008; Rengachary et al., 2009).

In the chronic phase, many right hemisphere damaged patients do not show neglect but extinction, i.e. difficulty in reporting a contralesional stimulus when it occurs simultaneously with a correctly reported ipsilesional stimulus. Extinction presumably results from the “winner-takes-all” functioning of the attention and awareness mechanisms within the parietal lobes (Driver and Vuilleumier, 2001). Although neglect and extinction frequently co-occur, several double dissociations have been described (Cocchini et al., 1999; Vossel et al., 2011) questioning whether extinction should simply be considered a “weak” expression of neglect in remission.

Although there is no doubt that the hallmark of neglect is the failure to attend to the contralesional hemispace, several studies have shown that additional deficits, not attributable to a spatial bias (i.e., non-lateralized), are associated with impaired processing of the contralesional hemispace (Husain and Rorden, 2003; Corbetta and Shulman, 2011, for review). Indeed, neglect patients may present several additional deficits, such as a lack of awareness of their impaired spatial processing (Karnath and Rorden, 2012), visuospatial working memory impairment (Wojciulik et al., 2001), increased variance in line bisection (Bonato et al., 2008), an abnormally long attentional blink (Husain et al., 1997; see also di Pellegrino et al., 1998), and, more generally, reduced alertness and sustained attention (Robertson, 2001). I will now focus on the latter two characteristics.

Reduced arousal and vigilance are often associated with right hemisphere injury (Heilman et al., 1978; Yokoyama et al., 1987; Lazar et al., 2002) and can also interact with spatial deficits (Robertson et al., 1995, 1997; Malhotra et al., 2009; Corbetta and Shulman, 2011). This interaction may be critical for the pathogenesis and preferential right hemisphere lateralization of neglect (Robertson et al., 1997; Corbetta and Shulman, 2011).

Studies by Ian Robertson and his collaborators have shown that the link between neglect and sustained attention is so close that the rehabilitation of the latter leads to benefits in the former (Robertson et al., 1995; see also De Gutis and Van Vleet, 2010), and that the presence and level of sustained attention deficits in right brain-damaged (RBD) patients accurately predicts the presence of neglect (Robertson et al., 1997).

In short, RBD patients have “disproportionate problems with a cluster of non-spatial attentional capacities” (Manly, 2002). However, to directly address the aims of this research topic, it is unclear to what extent non-lateralized deficits are caused by unspecific effects due to the size and lateralization of the cerebral lesion (often quite massive in RBD patients with neglect), or are instead an intrinsic characteristic of the neglect syndrome. The influential review by Husain and Rorden (2003), often quoted to support the view that neglect syndrome would be characterized by non-lateralized deficits, in fact cautiously suggested that non-lateralized deficits are often associated with neglect, but did not state that these deficits are to be considered an intrinsic characteristic of neglect.

One methodological caveat worth discussing in this context is that the presence of non-spatial deficits in neglect patients might be, at least in some studies, due to the presence of more severe, yet non-specific, cognitive impairments within the neglect group.

This could be due to two potential selection biases in studies where brain-damaged patients are assigned to a group according to the presence of a single clinical criterion. The first bias is that (severely impaired) left brain-damaged patients with large lesions and severe aphasia are systematically excluded from testing protocols because their comprehension deficits do not allow clinical or experimental tests to be performed. This may result in the selection of more severely impaired RBD (vs. left BD) patients (Kertesz et al., 1979; De Renzi, 1982). The second potential selection bias comes from the fact that a group of patients selected based on the presence of a specific deficit (e.g., neglect) present with more severe general cognitive impairments (as empirically indexed by lower scores neuropsychological tests and overall slower reaction times) than the complementary group (e.g., patients without neglect) derived from the same sample (Bonato et al., 2012b). In turn, this bias may result in the selection of more severely impaired neglect (vs. non-neglect) patients.

From a clinical perspective, the studies by Robertson et al. (1995, 1997) clearly showed that the diagnosis and rehabilitation of neglect can be more effective if the role played by sustained attention is taken into account. In addition, it is well established that the ubiquitous slowing down observed after right hemisphere damage is more prominent in the presence of neglect (Schürmann et al., 2003) and can be detected also when non-spatial aspects are investigated (see Howes and Boller, 1975; Samuelsson et al., 1998). This slowing down, however, cannot be *a priori* taken as indexing impairments in sustained attention rather than general, unspecific, impairments. The question thus becomes whether non-lateralized aspects of neglect are relatively independent from the severity of general and specific impairments suffered by patients or whether they are instead closely connected with spatial impairments. Only a few studies attempted to unravel these tangled issues (e.g., Hjaltason et al., 1996; Samuelsson et al., 1998). Even assuming that a genuine dissociation between neglect severity and non-specific impairments emerged in these two studies, this cannot be generalized by default to all studies where neglect patients show a more prominent slowing of processing.

Given that severe cognitive impairments often result in sustained attention deficits, caution is mandatory when considering as causal the several correlations between the indexes of neglect and sustained attention.

## Specific/Non-Specific Cognitive Resources in the Attentional Processes of Healthy Participants and Neglect Patients

Regardless of whether it should be considered solely spatial in nature, neglect is by and large considered an attentional disorder. There are several definitions of attention and every theory on “attention” aims to characterize one of several attentional processes, from visuospatial orienting to executive functions. Posner (1980) made an influential proposal, mostly focused on the characteristics of attentional orienting in visual space. He adopted a simple and informative method for dissociating components of visual attention (see Figure 2). Despite the presence of a more complete model encompassing the mechanisms for alerting/sustained attention (Posner and Petersen, 1990) most of the studies that adopted Posner’s cueing paradigm focused on clearly defining the differences between voluntary and automatic orienting, where unspecific non-spatial attentional resources are of little importance. This approach was very fruitful and showed that automatic (exogenous) components of attentional orienting in neglect are more impaired than voluntary ones (Losier and Klein, 2001; Bartolomeo and Chokron, 2002).

In contrast, other theoretical frameworks designed to account for dual-task performance suggested the crucial role played by task demands (see Kahneman, 1973, for a classic account) to determine the performance outcome. In particular, Wickens (2002) focused on dual-task interference and assigned an important role to non-specific cognitive resources for performance. According to him the term “resources” indicates, by definition, something which is limited and can be allocated. He distinguished between the characteristics of “resource demand” determined *a priori*, such as in the case of different experimental conditions, or *a posteriori*, by analyzing performance through subjective ratings, physiological measures, and, also by behavior. This differentiation allows to address the issues of resources, task difficulty, and resource demands and limits the risk of incurring in circular issues. It avoids stating that a task is more demanding because it results in slower responses and more errors, while maintaining that a task presents slower responses and more errors because it is more demanding. La Berge and Brown (1989) also highlighted the important role played by cognitive resources in attentional performance. Although their approach mainly focused on shape identification, it also addressed the debate on the mechanisms underlying orienting of spatial attention. They argued that attention operates in space not as a moving spotlight-model but as a gradient model of processing resources according to which peaks of resources and processing efficiency are formed at the location in space where attention is directed. To our knowledge the stances of Wickens and of Laberge and Brown are mostly confined to studies in ergonomics and experimental psychology, and have not been systematically addressed by studies conducted on brain-damaged patients.

A recent approach relevant for our purposes is the “load theory of attention” (Lavie et al., 2004), where the influence of lateralized distracters depends on the level and type of load required by the task. High perceptual load would reduce distracter interference, whereas working memory load or dual-task load would increase distracter interference. The load theory distinguishes between a perceptual selection mechanism and a cognitive control mechanism. The first reduces the perception of distracters in situations of high perceptual load that “exhaust perceptual capacity in the processing of relevant stimuli,” whereas the second is thought to reduce interference from perceived distracters as long as cognitive control functions are available to prioritize the tasks (i.e., under low cognitive load). This theory integrates the debate on the locus of selection of irrelevant information (early vs. late) into a unique, flexible system which processes (or does not process) lateralized distracters according to the amount and nature of the resources required by the task. It has been implemented directly to assess the performance induced by ipsilesional distracters in neglect (Lavie and Robertson, 2001; Snow and Mattingley, 2008).

Finally, two accounts of neglect are worth mentioning for the purposes of this paper.

The first suggests a key role for arousal, alertness, and sustained attention deficits in determining impaired visuospatial performance (Robertson, 2001), to the extent that the latter can be improved when on-the-spot alertness is increased (Robertson et al., 1998) or when it undergoes specific training (Thimm et al., 2009).

A second influential model of neglect focuses on the occurrence of three components: an initial, automatic orienting of attention toward the ipsilesional side; a general non-directional attentional deficit, and an impairment in reorienting attention toward the contralesional side (Karnath, 1988). According to this proposal, persisting deficit in the first two components would account for the residual deficits found in patients who have otherwise regained some contralesional orienting abilities.

After this brief theoretical overview, evidence will be presented showing that impairments in the processing of the contralesional hemispace can be detected more sensitively by tasks that do not allow any compensation.

## Computer-Based Testing and Increased Task Demands Result in Awareness Deficits for Contralesional Hemispace

### Computer-based testing detects hidden contralesional deficits

The adoption of computer-based testing is a promising solution for neglect assessment (Schendel and Robertson, 2002, for review) because it is potentially more sensitive than paper-and-pencil tests in detecting slowed processing of contralesional hemispace. Computerized assessments allow presenting patients with stimuli of brief durations and recording response latencies with a millisecond precision and can be adapted to the individual degree of impairments (List et al., 2008).

There is a long tradition of computer-based studies that have assessed the performance of RBD patients in computer-based detection tasks, which typically require patients to press a response key when a lateralized target is perceived. These computer-based approaches often highlighted slower responses for targets appearing in the contralesional hemispace, also in patients without evidence of neglect on paper-and-pencil tests. For instance, the difference between the detection of validly cued left vs. right targets is biased toward the ipsilesional hemispace in chronic RBD patients, even in the absence of neglect (Posner et al., 1984; Losier and Klein, 2001) and even when patients without neglect or extinction are included in the sample (Friedrich et al., 1998). These studies mainly focused on contralesional slowing rather than on omission rate because targets of relatively long durations (e.g., never shorter than 2 s according to Losier and Klein, 2001) were presented, whereas shorter durations are required to obtain a consistent number of omissions.

It may be argued that the disadvantage found in contralesional targets detection may be due to biased orienting in valid trials. However, this explanation can be refuted by the results of studies where RBD patients without neglect at clinical testing were required to detect single, brief light-emitting diode (LED) flashes occurring at several eccentricities (up to 40°; Smania et al., 1998; Marzi et al., 2002). Despite the fact that there were no cueing procedures (and therefore, valid trials) this LED-based testing procedure allowed detecting severe contralesional slowing and omissions. In particular, when the same patients were presented with stimuli that always appeared in the same location within each block, their performance returned to normal (Marzi et al., 2002). The method adopted by Marzi et al. (2002) and by Smania et al. (1998) required to simultaneously monitor several spatial positions where the target could potentially appear. It is plausible that the sensitivity of this device in detecting attentional biases for contralesional hemispace therefore derives from a high recruitment of monitoring resources, due to both the wide range of locations where the target could appear and to its brief duration. Indeed, spatial predictability improves target detection performance (Geng and Behrmann, 2005). From this perspective, it seems plausible that the deployment of resources for spatial monitoring would result in an increase in the cognitive load required by the task. The recruitment of visuospatial resources might then, in turn, hamper the implementation of compensatory strategies and allow subtle deficits to emerge. This testing method is sensitive enough to detect signs of contralesional slowing in both left and RBD patients (Smania et al., 1998). Target duration of computer-based testing can be calibrated individually in order to avoid floor and ceiling effects. This procedure allows analyzing, for each patient, both RTs and omission rates and is particularly suitable for exploring the effects of changes in the task instructions, while keeping the same stimuli across the different tasks (Vuilleumier and Rafal, 2000). Patients with right hemisphere damage are also particularly slow at detecting a contralesional target when it is preceded by an ipsilesional cue. In the seminal study by Posner et al. (1984) this “disengage deficit” occurred despite the fact that the study included several patients with mild or no neglect according to clinical testing, based on easy everyday activities, and one case without extinction at standard finger confrontation testing. The disengage deficit can persist, in the absence of neglect on paper-and-pencil tests, for several years after lesion onset (Friedrich et al., 1998). It also emerges when attention is oriented rightwards by a non-predictive arrow cue presented at fixation (Bonato et al., 2009).

Most, if not all, the detection studies performed with brain-damaged patients (Losier and Klein, 2001, for review) focused on theoretical issues and did not highlight the possible clinical (i.e., diagnostic) usefulness of these tasks. Contralesional slowing (or omissions when brief target durations are adopted) commonly occur in computer-based (but not in paper-and-pencil) experimental tasks, even when the non-neglect group is based upon performance on sensitive and complex diagnostic batteries as the Behavioral Inattention Test (BIT; Wilson et al., 1987), which is considered the “gold standard” for neglect diagnosis (Halligan et al., 1989).

Increased visual (Lavie et al., 2004; Dell’Acqua et al., 2006) or visual and auditory (Webster and Haslerud, 1964) load in healthy participants hampers processing at peripheral locations. A number of studies with RBD patients manipulated visual demands at fixation. The mere presence/absence of a fixation point can determine whether a brain-damaged patient will show neglect, hemianopia, or both (Walker et al., 1991; Müller-Oehring et al., 2003). Furthermore, RBD patients show a bias in disengaging attention from fixation (Posner et al., 1984; Ptak et al., 2007).

Crucially, the deployment of attention in brain-damaged patients may be differentially affected in the two hemispaces by increasing the attentional resources deployed at fixation. Increasing perceptual demands at fixation (e.g., by asking the discrimination of a shape) can result in an asymmetric reduction of spatial performance with a significant “shrinkage” of the contralesional hemifield in RBD patients without neglect (Russell et al., 2004). A similar manipulation resulted in a more efficient rejection of ipsilesional distracters in neglect patients, as predicted by the load theory of attention (Lavie and Robertson, 2001; but see Snow and Mattingley, 2008). Two recent studies of RBD patients with left neglect (Vuilleumier et al., 2008; Eramudugolla et al., 2010) have confirmed that increased load at fixation deeply affects contralesional hemispace processing (see also Maravita et al., 2007). The f-MRI study by Vuilleumier et al. (2008) demonstrated that increased load at fixation can reduce or even eliminate brain activations selectively for (ipsilesional) visual areas which process the opposite hemispace. Instead, Eramudugolla et al. (2010) showed that impairments for the contralesional hemispace exerted by increased load at fixation are so strong that they are relatively unaffected by prismatic adaptation.

In summary, several studies unite to show that brain-damaged patients without neglect on paper-and-pencil tests are slow to detect computer-presented contralesional targets, and that increased visual demands at fixation can result in the complete disruption of contralesional processing.

### Clinical relevance of computer-based, sensitive testing

Only a few studies have examined the clinical implications of how neglect can (re)emerge with computer-based presentation.

In a seminal study, Anton et al. (1988) presented a group of right hemisphere damaged patients with a series of unilateral or bilateral lights appearing on a semicircular array covering a wide visual angle, and three paper-and-pencil tests. Occupational therapy scores were also collected. The sample was then categorized according to the presence of neglect in the computerized test (54%), in the standard tests (20%), and in the occupational therapy test (28%). In other words, the light detection task resulted to be more sensitive than the standard clinical measures (see also Beis et al., 1994; Eschenbeck et al., 2010).

**Figure 1:**
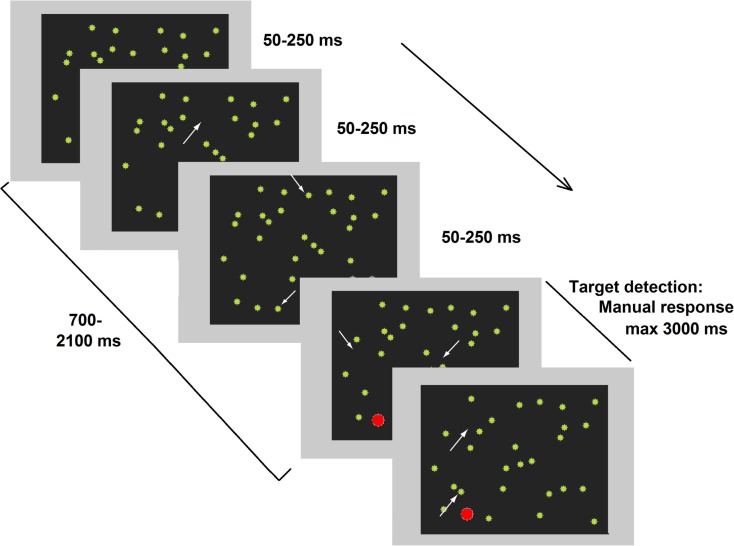
**Representative frames of one trial of the dynamic Starry Night Test (SNT)**. White arrows (not present in the real test) point to spatial positions where a distracter (green dot) appeared or disappeared along the trial. The target (in red) was embedded in the continuously changing background. Adapted from Deouell et al. (2005), image not to scale.

More recently, a compelling study (Deouell et al., 2005) directly compared results from the computer-based Starry Night Test (SNT, see Figure 1) and paper-and-pencil tests (BIT). A higher sensitivity emerged in the SNT compared the BIT when assessing each patient’s individual performance. In the SNT, relatively brief targets can appear in several spatial positions. As previously noted for the studies by Marzi et al. (2002) and Smania et al. (1998) spatial uncertainty plausibly deploys attentional monitoring resources and hampers the implementation of compensatory strategies. Moreover, in the SNT, the presence of distracters does not allow patients to respond (key press) as soon as something appears on the screen but forces them to identify the target before responding. Crucially, Deouell and collaborators also described in detail the deficits shown in everyday life by two patients whose neglect was only evident in the SNT (see also Erez et al., 2009).

**Figure 2:**
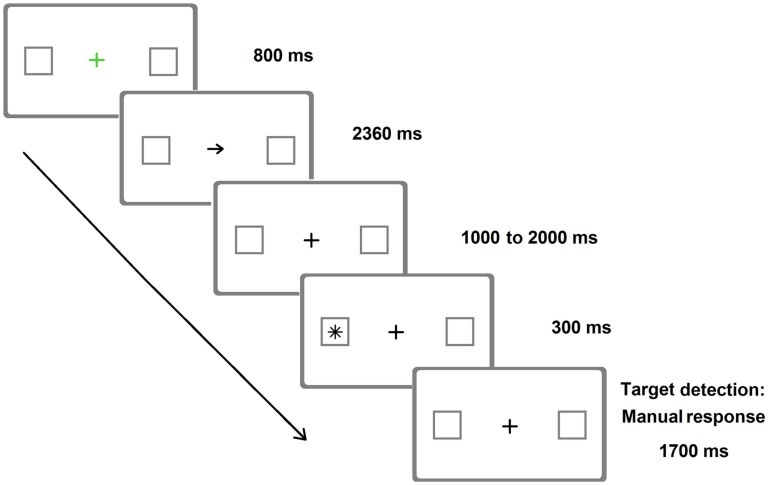
**Representative frames of one (invalid) trial of the Posner Cueing Paradigm resembling the version adopted by Rengachary et al. (2009)**. Arrows predicted the location of appearance of the target on 75% of the trials. The image is only indicative of the stimuli adopted and is not to scale.

As already mentioned, Posner-like detection tasks can also be more sensitive than paper-and-pencil tests in unveiling neglect (e.g., Friedrich and Margolin, 1993). This occurs not only in the chronic but also in the acute phase (Rengachary et al., 2009). Relatively brief target durations (Figure 2) increase the sensitivity of these tasks (Rengachary et al., 2009, 2011, where a variant of the Posner paradigm with very long SOAs was adopted).

Non-spatial characteristics of a task can also increase the amount of resources required for performance. For instance, the mere introduction of trials where no response is required can increase the left-right asymmetry in RBD patients without neglect (Bartolomeo, 2000). This suggests that apparently intact RBD patients tested by previous studies often presented hidden neglect and were able to compensate for their spatial bias through the task (Plummer et al., 2003; see also Appelros et al., 2003; Behrmann et al., 2004).

The connection between subclinical neglect and the demanding tasks which often characterize everyday life became even more evident in a visual dual-task study where a sample of stroke patients were divided into two groups according to whether they were/were not still driving at the time of testing (Marshall et al., 1997). Despite intact performance at paper-and-pencil tests for neglect assessment, the better dual-task performance obtained by the drivers group was interpreted as though these patients were more efficient in dealing with complex visuospatial tasks requiring intact divided attention skills. A simple yet challenging driving environment, where lateral items had to be detected, was reproduced in an experimental study where more patients were classified as affected by neglect according to reaction time asymmetry in the “driving” task than the BIT criteria (van Kessel et al., 2010). Within the RBD group, patients with both RT asymmetries and pathological BIT scores showed longer ipsilesional RTs than patients with RT asymmetries only. Once again, neglect symptoms were detected when more demanding tests were conducted on patients who were able to compensate for their lateralized deficit in paper-and-pencil tasks.

To our knowledge only Peers et al. (2006) reported that, contrary to the previously discussed evidence, an increase in task demands result in a rightward shift of attention, independent from lesion lateralization.

### Detecting unrecovered neglect

Some studies addressed the clinical potential of tasks that do not allow compensation by exploring whether these testing procedures can still detect neglect in the specific case of patients who had a clinical history of neglect and then recovered. Severe impairments of the contralesional hemispace in (apparently) recovered patients can re-emerge if sensitive testing procedures and scoring criteria are implemented (Campbell and Oxbury, 1976). RBD patients without signs of neglect exhibit an early rightward orienting of attention when identifying complex stimuli such as overlapping figures (Gainotti et al., 1991). This supports the view of Karnath (1988) who maintains that a strong tendency toward rightward orienting is a core deficit in chronic neglect. Once again, the RBD group in Gainotti et al. (1991) may have included neglect patients who were, in fact, able to inhibit their initial rightward orienting after its occurrence, and to redirect their attention toward the left (i.e., to compensate for their deficit) in standard clinical tests, characterized by less complex stimuli and less sensitive, accuracy-based, scoring criteria.

Bartolomeo (1997) studied the individual performance changes within a computer-based detection task. He found that (apparently) recovered neglect patients showed longer RTs to left-sided visual stimuli than to right-sided stimuli at the beginning of the test, but fell within the controls’ range by the end of the test. Patients with mild albeit hidden neglect thus seemed to be rather effective in recruiting attentional resources to compensate for their deficits when performing tasks engaging visuospatial attention. It may seem that this finding is in contrast with the hypothesis of a sustained attention deficit in neglect. Instead, it supports the idea that only neglect patients with severe general/sustained attention deficits are unable to compensate for their spatial deficits. The ubiquity of these compensatory strategies makes it difficult to clarify whether the majority of patients who seem to spontaneously recover from the spatial biases of the disorder in the first phases, have genuinely recovered or are, in fact, implementing corrective compensatory (voluntary) strategies (Robertson and Manly, 2002).

As already mentioned, the SNT is also successful in detecting patients who had apparently recovered from neglect. It was sensitive enough to detect slower RTs for contralesional (as compared to ipsilesional) stimuli in a RBD patient tested 12 years after a stroke, who had severe but hidden deficits in everyday life, including a severe problem in driving as indicated by several crashes involving the left side of his car (Deouell et al., 2005).

As suggested by Campbell and Oxbury (1976), an accurate analysis of behavioral performance may also reveal that the recovery from neglect in several patients is only apparent. Post-acute stroke patients who were diagnosed with neglect and then re-tested on average about 5 months after stroke may show unimpaired performance on standard paper-and-pencil tests but mild impairments in their movements (Goodale et al., 1990). While post-stroke patients had the same overall accuracy of their arm reaching movements of healthy controls, kinematics revealed significant rightward deviations in their trajectories that were only corrected in the final (pre-target) stage. Patients who had (apparently) recovered from left neglect also showed biased visual exploration with a shift toward the right side of items (Mattingley et al., 1994; see also Pflugshaupt et al., 2004). In cancellation tasks, patients with left neglect show several markers of biased performance (e.g., rightward starting point, slowness, increased speed variability, and incoherent organization), which typically are not taken into account by standard, accuracy-based, criteria (Manly et al., 2009). A careful assessment of these performance details also confirmed the presence of visuospatial impairments for those patients whose scores were borderline (around the cut-off) on paper-and-pencil tests and for which the appropriateness of the neglect diagnosis can be questioned (Manly et al., 2009).

### Lack of sensitivity in neglect diagnosis: Sensitive diagnosis requires difficult tasks

It is a truism to maintain that a more difficult task results in a worse performance and that only difficult tests achieve higher diagnostic sensitivity. Indeed, several studies adopting cancellation tasks have shown that the performance of neglect patients decreases when attentional demands are increased and more complex visual searching strategies are implemented (Rapcsak et al., 1989; Eglin et al., 1991; Aglioti et al., 1997; Sarri et al., 2009). Surprisingly enough, the implementation of difficult tasks to increase the sensitivity of diagnostic tests is far from being a standard in assessing neglect and extinction.

On the contrary, several clinical studies in different research domains have shown that difficult tasks result in more sensitive diagnosis and allow to infer performance in everyday contexts. For example, research in the field of fall risk in older people has shown that dual-task performance (e.g., walking combined with a simultaneous cognitive task), hampers motor performance, particularly when cognitive deficits are also present (Camicioli et al., 1997). A second example comes from the study of patients with cirrhosis, which shows that a highly demanding visuospatial task (i.e., performing a sustained attention task on briefly presented letters) is sensitive in detecting the presence of minimal hepatic encephalopathy (Amodio et al., 2010). Therefore, it seems appropriate to implement more demanding visuospatial tasks in order to obtain a more sensitive diagnosis of neglect, a disorder which in itself is visuospatial.

Apart from its theoretical consequences, the misdiagnosis of neglect raises a number of important clinical implications since patients in the chronic phase may be allowed to return to their pre-morbid activities where they may be at risk (driving, road crossing, and use of dangerous objects/devices). Experienced clinical neuropsychologists know that paper-and-pencil tests can detect only moderate-to-severe forms of neglect (Barrett et al., 2006; Buxbaum et al., 2004) and do not allow deducing a patient’s disability in natural settings (Deouell et al., 2005; Hasegawa et al., 2011). Nonetheless, these tests are still considered the “state of the art” for diagnosing contralesional awareness deficits, despite several studies showed that, in the chronic phase, computer-based testing is the best option for obtaining a more sensitive diagnosis of neglect (Friedrich and Margolin, 1993; Schendel and Robertson, 2002; Deouell et al., 2005; Rengachary et al., 2009; Bonato et al., 2010, 2012a,c).

Moreover, although studies by Bartolomeo (1997, 2000) suggest a positive answer, the question as to whether a non-specific (non-visual) increase in the amount of attentional resources worsen neglect, and to whether this might also occur in patients with intact performances on paper-and-pencil tests have been scarcely addressed so far. Robertson and Frasca (1992) directly addressed the first issue by showing that the performance of left neglect patients in cancellation and reaction time tasks can be modulated by different engagements of working memory in a concurrent task (e.g., from an easy task like counting forward to a hard one like counting backward by threes from 100). In their study some (but not all) neglect patients showed a peculiar contra-ipsilesional increase in the detection of lateralized targets when performed with a simultaneous attentionally demanding concurrent task (i.e., counting backward in threes from 100). The study adopted a multiple single-case approach where each patient’s individual performance could be tested for asymmetries and dual-task modulation. Nevertheless, the experimental paradigm did not highlight an increased bias in patients without neglect. If volitional orienting plays an important role in functional recovery from neglect, a re-emergence of the contralesional deficit under challenging dual-task conditions (Robertson and Manly, 2002) could be predicted. We empirically confirmed this prediction.

### Combining computer-based presentation with demanding tasks

We recently combined brief stimuli presentation with resource-demanding tasks, namely two characteristics that have been shown to maximize the possibility to detect contralesional omissions in a multiple single-case study of four post-acute (1–2 months from stroke) RBD patients (Bonato et al., 2010).

**Figure 3:**
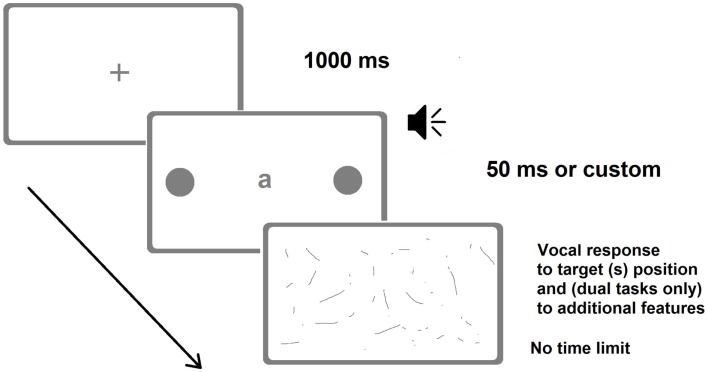
**Representative frames of one (bilateral) trial of the Dual-Task Paradigm in the version adopted by Bonato et al. (2012a)**. There were also trials with a single unilateral target. The loudspeaker indicates the auditory presentation of a number. In the last screenshot (response collection) patients verbally reported either the position of the target(s) only (in the single-task condition) or the identity of the letter and the position of the target(s) (visual dual-task) or counted twice by two from the number they heard and then reported the position of the target(s) (auditory dual-task). Image not to scale.

Patients were first tested for the presence of neglect with the BIT and then for the presence of extinction with the finger confrontation procedure. One of them had neglect and extinction, whereas the remaining three had no signs of neglect and only one of them showed signs of extinction. Patients were required to verbally report the position of briefly presented unilateral and bilateral targets (Figure 3). Target duration was individually determined by means of a calibration procedure performed before the experiments (Vuilleumier and Rafal, 2000). Upper and lower limits were set to 50 and 700 ms, respectively. Within the calibration procedure for each bilateral trial (of a given duration) the accuracy was calculated online and determined the duration of the subsequent trial, which was increased or decreased by 35 ms, depending on whether the patient extinguished or correctly reported the contralesional target, respectively. The calibration procedure yielded individual target durations between 50 and 650 ms. For control participants, target duration was set at the minimum allowed (50 ms).

The average extinction rate for bilateral trials dramatically increased under dual-task conditions from 18.4% in the single task to 90% in the visual dual-task and to 84% in the auditory dual-task. Impairments for contralesional space processing, thus, emerged as soon as the quantity of attentional resources available for performing the task were reduced, regardless of the nature of the concurrent task (i.e., visual vs. auditory). Two patients, despite the absence of neglect on paper-and-pencil tests, omitted a significant number (30 and 80%) of single contralesional targets (i.e., they showed neglect), once again only under dual-task conditions. In contrast, the performance of healthy participants was symmetric and virtually errorless, and unaffected by the dual-task manipulation. A left brain-damaged patient without neglect, tested 4 months from stroke, also showed severe contralesional (this time, right) awareness deficits for single and double targets (neglect and extinction, respectively), under dual-task conditions only. The performance of healthy controls and of the left brain-damaged patient demonstrate that the spatial deficits found in RBD patients are genuinely contralesional and not due to an unspecific rightward shift that may occur under dual-task conditions (Peers et al., 2006 vs. Śmigasiewicz et al., 2010).

Bonato et al. (2010) explicitly focused on the bias resulting from manipulations in task difficulty and highlighted its diagnostic potential. It is worth reiterating that neglect severity is closely determined by the task at hand. Surprisingly enough, until our study, computer-based and demanding testing had never been coupled to assess the potential presence of neglect in patients whose contralesional awareness was apparently spared. Moreover, previous studies on patients (except Robertson and Frasca, 1992) assessed the effects of load manipulations in visuospatial modality only. As a result, it is difficult to disentangle whether the severe impairments in contralesional awareness resulting from an increase in demands at fixation were caused by an increase in the visuospatial load or by an unspecific recruitment of attentional resources, or the combination of both. Consequently, a crucial and novel aspect of our findings is that, regardless of whether visual or non-visual processing resources are recruited, and the extent to which the two manipulations can be considered similar, both dual-task manipulations can have a detrimental effect on awareness. Finally, the choice to adopt a multiple single-case approach (and statistics) was fundamental to allow us to monitor performance at the individual level (Robertson and Frasca, 1992; Deouell et al., 2005).

The lack of a systematic discussion about the need to implement more sensitive testing procedures may be due to the absence of strong connections between everyday life performance and subtle awareness deficits that emerge in a computer-based task. Researchers may believe that deficits detected by computer-based testing procedures are so mild that they do not exert any effect on everyday performance. Moreover, the few studies assessing patients’ behavior in everyday life generally rely on standard instruments to obtain an “ecological” assessment (e.g., FIM, Barthel index, Bergego scale). To our knowledge only a few studies (e.g., Deouell et al., 2005; Hasegawa et al., 2011) described in detail the impairments shown by chronic patients without neglect according to paper-and-pencil tests in complex, truly ecological, everyday settings. The main disadvantage of the FIM, Barthel and Bergego scales is that the resulting scores only allow quantifying disability in easy tasks such as eating or dressing, but do not appear to be precise enough to detect subtle neglect in complex everyday life activities, and lack scores related to dual-task performance (but see Eschenbeck et al., 2010 for a more sensitive neglect-related ADL assessment). Additionally, they do not clarify whether contralesional performance is impaired because of motor or attentional deficits when patients, as commonly occurs, have concurrent motor deficits. In order to answer both criticisms, we (Bonato et al., 2012a) recently performed a longitudinal investigation on the deficits shown by GB, a 63-year-old woman who, after a stroke affecting most of the territory of her right middle cerebral artery, showed no motor impairment and presented normal performance on paper-and-pencil tests for neglect (see the Tutorial in the Supplementary Material). We tested her with computer-based, resource-demanding dual-task procedures and repeated ecological observations at home, for more than 6 months after her discharge from the hospital. Surprisingly enough, both computer-based and observation-based approaches highlighted severe difficulties in contralesional hemispace processing which selectively emerged under dual-task conditions, not only in the computer-based paradigm (she neglected almost all contralesional targets in the first testing session) but also in everyday life (with repeated bumping into objects on her left).

The uncommon absence of any motor deficits (Azouvi et al., 2002) allowed us to rule left leg/arm weakness as a potential explanation for her accidents involving bumping into objects on her left and to ascribe to neglect her impaired performance for contralesional hemispace found in everyday life contexts. Longitudinal testing allowed us to detect the spontaneous remission of GB’s deficits over time, which began from the easiest conditions (single task) and continued to intermediate difficult conditions (dual task, single stimulus), resulting, after more than 1 year, in the sole persistence of extinction for 1/3 of the trials performed under dual-task conditions only (Bonato et al., unpublished data).

She was also presented with several cancellation tasks (Bonato et al., 2012a), see Videos 1–3 in the Supplementary Material. She was very accurate and relatively fast, although her starting point, an index of subclinical neglect (Azouvi et al., 2002) was consistently located in the right half of the page. Her performance on the TMT-A was normal, whereas her TMT-B performance was very slow (Videos 4 and 5). This confirmed that she suffered from severe visuospatial impairments under dual-task (in the case of the TMT-B: task shift) conditions, exacerbated also when two spatial positions had to be monitored to determine the order of appearance of two targets (Video 6). Cued-detection tasks (e.g., Posner, 1980; Rengachary et al., 2009) revealed a persisting contralesional slowing in target detection which was, however, not coupled with a significant number of contralesional omissions or with a disengage deficit when the test was performed in the chronic phase.

In the same study we also tested, with the same computer-based dual-tasks, five post-acute right stroke patients (one with left neglect and four without neglect; four with extinction rate of ≤35% at finger confrontation) and one healthy participant (sex and age-matched with GB). Contralesional extinction and omissions also dramatically emerged for these patients, confirming that subclinical awareness deficits in post-acute stroke patients are more the rule than the exception. Across the same group, the effects of different target durations were also compared across patients. Each patient was presented both with a customized target duration – calculated using the “calibration procedure” described above (resulting range of 50–600 ms) – and with the minimal (50 ms) target duration. These two conditions succeeded, respectively, in maximizing the emergence of (i) contralesional awareness deficits under dual-task conditions and (ii) contralesional awareness deficits regardless of dual-task manipulations. In no case did the BIT (for neglect) or the finger confrontation procedure (for neglect/extinction) detect a deficit that did not emerge under dual-task conditions. In contrast, our dual-task succeeded, both at an individual and group level, in highlighting deficits that were much more severe than those detected by standard clinical testing. This was maximally evident when target duration was as short as 50 ms, with an average left omission rate above 80% for bilateral targets and around 50% for left unilateral targets.

In a third study (Bonato et al., 2012c) we grouped the data from ten RBD patients who had been presented with the shortest (50 ms) target duration in our computer-based, resource demanding task. We directly compared their omission rates when performing the BIT cancellation subtests and when performing the computer-based tasks. The difference in performance found was, once again, striking. Across all cancellation tests, only 7% of omissions for left targets vs. 5.5% for right targets emerged (i.e., no left-right difference was observed). In contrast, under dual-tasks, computer-based conditions, patients omitted 70% of unilateral targets on the left and 4.5% on the right (i.e., a significant left-right difference was observed).

Our approach therefore allows to couple a bottom-up (e.g., long vs. brief stimuli presentation) with a top-down manipulation (e.g., single vs. dual-task condition). Several patients already showed impaired contralesional performance under single-task conditions and custom target duration. The deficits became even more evident when the presentation time was reduced, and the awareness for contralesional hemispace further decreased under dual-task conditions (Bonato et al., 2012c). Our data were interpreted by maintaining that the reduction in the presentation time and the introduction of a concurrent task both contributed to the recruitment of attentional resources, which in turn resulted in contralesional awareness deficits.

Even though both manipulations seemed to converge in increasing awareness deficits selectively for contralesional hemispace, in future they may be useful for providing different information on the individual characteristics of the awareness deficit of the tested patient. Indeed, some patients may be more sensitive to dual-task manipulations whereas some may be more sensitive to reduced presentation time.

Although fully addressing the complex relation between neglect and extinction goes beyond the aims of this review, our results reliably show more severe extinction than neglect (Bonato et al., 2010, 2012a) at a group level. At the same time, however, it is worth mentioning that the only left hemisphere damaged patient we tested (Bonato et al., 2010) presented with more severe neglect than extinction, as if the presence of a left target was, in his case, facilitating the detection of a right, synchronous target. Whether this phenomenon is a general characteristic of left brain-damaged patients and/or whether it can also be found in RBD patients remains unanswered.

Further evidence is also needed to clarify differences between dissimilar dual tasks. At present, group data show a similar modulation in the number of neglected targets (Bonato et al., 2012c) regardless of the version of the dual task adopted. At an individual level, however, some patients seem to be more affected by a specific load manipulation (Bonato et al., 2010, 2012a). If our interpretation of hampered performance as a function of attentional resources engaged by the task is correct, dual tasks with different levels of difficulty should result in better performance for the easier one.

Our results suggest that deficits in awareness emerge drastically in the contralesional hemispace when attentionally demanding tasks are performed and compensatory strategies cannot be implemented. Resource-demanding dual-tasks appear to be one of the best options available for detecting and monitoring the presence of awareness deficits from lesion onset over time (see Deouell et al., 2005; and Rengachary et al., 2009 for recent alternative, sensitive, computer-based assessment methods). A comparison with the average evolution of performance on standard tests is shown in Table 1. Our approach might be useful for neuropsychologists not only because it is sensitive but also because it is flexible and informative. It is flexible because it allows the use of different indicators according to the severity of the awareness deficits, the easiest conditions being single task and unilateral target presentation and the more difficult (and sensitive) being bilateral target presentation under dual-task conditions. It is informative because it allows identifying patients whose visuospatial performance in everyday life can be kept within the boundaries of normality by avoiding dual-task recruitments.

**Table 1:**
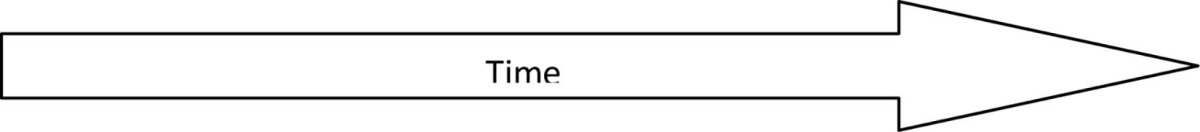
**A simple graphical representation of hierarchy of spatial impairments presented by a “typical” patient following a stroke of the middle right cerebral artery in the acute, post-acute, and chronic phases, respectively**.

Symptom	Acute phase (first days)	Post-acute phase (1 month)	Chronic phase (3–6 months)
Rightward gaze	Y	N	N
Left omissions: easy (e.g., no distracters) cancellation tasks	Y	N (but right starting point)	N (but right starting point)
Left omissions: difficult (e.g., with distracters) cancellation tasks	Y	Few/inconsistent	N (but right starting point)
Contralesional omissions at computer-based single tasks	Y	Several	N
Contralesional omissions at computer-based dual tasks	Y	Several	Y (Variable)
Contralesional extinction at finger confrontation	Y	Y	N
Contralesional extinction at computer-based dual tasks	Not possible to assess	Y	Y

*In the post-acute phase, computer-based dual tasks are more sensitive than cancellation tasks in detecting neglect. In the chronic phase, computer-based dual tasks are more sensitive than finger confrontation in detecting extinction*.

In summary, we (Bonato et al., 2010, 2012a,c) provided a concrete diagnostic tool, and confirmed that: (a) the degree of contralesional impairments was closely dependent on the amount of resources required by the task and (b) apparently spared contralesional awareness may simply reflect the general availability of attentional resources that just suffice to perform single tasks.

## Final Considerations

### Summary

We have reviewed evidence that:

– Neglect patients have non-lateralized deficits which interact with the severity of lateralized deficits and can enhance them (Husain and Rorden, 2003; Corbetta and Shulman, 2011). It seems very difficult to separate the overall role played by non-specific cognitive impairments (De Renzi, 1982; Bonato et al., 2012b).
– Computer-based detection tasks highlight contralesional impairments which are not detected by paper-and-pencil tests (Posner et al., 1984; Friedrich et al., 1998; Smania et al., 1998; Losier and Klein, 2001; Marzi et al., 2002; Bonato et al., 2009).
– The true impairment suffered by patients is revealed, in the chronic phase, only when attentional resources, otherwise implemented to contrast and compensate for the contralesional bias, cannot be effectively allocated (e.g., Bartolomeo, 1997, 2000; Marzi et al., 2002; Deouell et al., 2005; Rengachary et al., 2009; Bonato et al., 2010, 2012a; van Kessel et al., 2010; Hasegawa et al., 2011).
– These tasks can be useful for clinical (e.g., diagnostic) purposes (Deouell et al., 2005; Rengachary et al., 2009; Bonato et al., 2010, 2012a,c).

### Integration with the theoretical positions

The findings above supplement the theories of normal attention accounting for a crucial role of non-specific cognitive resources in dual-task performance (Wickens, 2002), as well as those claiming a gradient of resources in space (La Berge and Brown, 1989). With specific reference to the load theory (Lavie et al., 2004), we found that contralesional orienting/awareness can be hindered similarly and independently from whether attentional load is increased visually at fixation (Russell et al., 2004; Vuilleumier et al., 2008) or by a second task irrelevant to visuospatial processing (Bonato et al., 2010, 2012a). We highlighted the importance of compensatory strategies (Bartolomeo, 1997; Robertson and Manly, 2002) the persistence of extinction after neglect remission (Karnath, 1988) and the presence of a disengage deficit from fixation (Posner et al., 1984; Ptak et al., 2007).

### Taking advantage of the asymmetry in neglect

Studies that suggested an individual-level comparison took advantage of a peculiarity characterizing neglect syndrome: the possibility to use the ipsilesional hemispace performance of an individual patient as their own control (e.g., Robertson and Frasca, 1992; Deouell et al., 2005; in part also Bonato et al., 2010, 2012a). This simple approach maximizes sensitivity and can be summarized as follows: poorer performance in the contralesional as compared to the ipsilesional hemispace indicates neglect. In contrast, the diagnosis of neuropsychological impairments other than neglect requires a comparison with the performance of a sample of healthy controls. This comparison results in lower sensitivity because inter-individual variability must be considered. Nevertheless, many studies based the diagnosis of neglect on the comparison with the performance shown by a standardized sample (e.g., the cut-off scores of the BIT) or, less frequently, on the cut-off scores shown by the worst of the healthy participants (e.g., Stone et al., 1992).

It is worth highlighting, however, that this approach does not necessarily assume that the ipsilesional hemispace is intact in patients with neglect. In fact, a neglect patient’s performance for the ipsilesional hemispace is far from “normal.” We have already mentioned several studies suggesting that disengaging from both ipsilesional and central cues is particularly difficult for RBD patients (Posner et al., 1984; Russell et al., 2004; Ptak et al., 2007). Considerable evidence suggests that attentional orienting toward the ipsilesional hemispace is characterized by slower and more error-prone detection of targets within the less eccentric ipsilesional positions (Smania et al., 1998). Extinction itself can be seen as an indicator of pathological reflexive orienting toward the “good” hemispace (but see di Pellegrino et al., 1997). At the same time, however, increased severity of neglect is coupled with slower reaction times for ipsilesional stimuli (Bartolomeo and Chokron, 1999). Finally, in several clinical tests (e.g., cancellation tasks), the performance of neglect patients is often characterized by perseverations (e.g., repeated marks on the same target), typically more evident for the most ipsilesional items (Ronchi et al., 2009) and potentially interacting also with deficits of monitoring/executive functions.

### Pros and cons of computer-based testing paradigms

Apart from high sensitivity, computer-based tests have several additional advantages; short administration time, low cost, and the possibility to easily modify and control the characteristics of the stimuli. Nonetheless, they also have some disadvantages. Firstly, the specific programs are currently not available from software companies^1^ and, therefore, their implementation requires specific software allowing for brief presentation time, RT recording, and some basic programming and statistical skills for calculating the individual statistics. Moreover, they are not suitable if the patient has hemianopia. In addition, they cannot be used to test for the presence of neglect in spaces other than the peripersonal one, although this limitation also holds for paper-and-pencil tests.

### Challenges for future studies

Future studies are required to increase both the theoretical and clinical relevance of tasks where no compensation is allowed. Their theoretical relevance could be increased by better defining the role played by general cognitive impairments in determining the performance of a single patient. Their clinical relevance can be increased by addressing three main questions. One relates to the incidence of subclinical neglect and to the factors resulting in the implementation of compensatory strategies in neglect patients. This question could be primarily answered by testing larger samples of patients. A second question regards the sensitiveness of these methods in disability prediction. This could be answered by implementing instruments to quantitatively determine performance across several, highly demanding, everyday life tasks. Within this specific domain, it would be interesting to explore, by analogy, whether extinction in computer-based tasks is coupled with contralesional impairments in complex environments when several ipsilesional distracters are presented. The third question relates to understanding which approach, among the few options available, is more sensitive (e.g., Bonato et al., 2010, 2012a vs. Deouell et al., 2005 vs. Rengachary et al., 2009). Regardless of whether computer-based testing is used, it seems important that future studies take advantage of the contra-ipsilesional comparison to obtain more sensitive tests.

The last step, and potentially the most difficult one, would involve implementing and testing successful rehabilitation procedures, which could even adopt a complex paradigm similar to the one used for the diagnosis. As noted elsewhere (Erez et al., 2009; Bonato et al., 2012a), only by coupling effective rehabilitation procedures with sensitive assessment it is possible to guarantee that any potential improvements in a patient’s performance are captured by the testing methods. More sensitive instruments will help to determine the most effective solutions to reduce impairment and disability in patients affected by this syndrome, which fascinates researchers but is a major obstacle in a patient’s steep and long road back to recover autonomy (Katz et al., 1999).

## Conflict of Interest Statement

The author declares that the research was conducted in the absence of any commercial or financial relationships that could be construed as a potential conflict of interest.

## Supplementary Material

The Supplementary Material for this article can be found online at http://www.frontiersin.org/Human_Neuroscience/10.3389/fnhum.2012.00195/abstract

## Footnotes

^1^Neuropsychologists interested in receiving and developing the E-prime script adopted for stimuli presentation in our studies can write to mario.bonato@unipd.it
